# Novel dual-fluorescent flow cytometric approach for quantification of macrophages infected with *Leishmania infantum* parasites

**DOI:** 10.1017/S0031182021001530

**Published:** 2022-01

**Authors:** Zeynep Islek, Mehmet Hikmet Ucisik, Fikrettin Sahin

**Affiliations:** 1 Department of Genetics and Bioengineering, Faculty of Engineering, Yeditepe University, İnönü Mah., Kayışdağı Cd. No:326A, 34755 Ataşehir/Istanbul, Turkey; 2 Department of Pharmaceutical Technology, Faculty of Pharmacy, Istanbul Health and Technology University, Seyitnizam, Mevlana Cd. No: 85, 34015 Zeytinburnu/İstanbul

**Keywords:** Detection of *Leishmania* infection, flow cytometry, fluorescent assay, *Leishmania infantum*, macrophages, PKH dyes

## Abstract

Flow cytometry analysis emerges as an alternative methodology to microscopy for determination of the *Leishmania*-infection rates of macrophages. Various flow cytometric approaches have been established for the quantification of *Leishmania* parasites within host cells, labelled either directly fluorescent dyes or indirectly with fluorescently conjugated antibodies. Although these techniques allow accurate quantification of infection, they fail at detection of non-infected macrophages specifically. This study introduces a new flow cytometric approach for the determination of infection rates of macrophages infected by *Leishmania infantum* parasites. Prior to infection, J774A.1 macrophages and *L. infantum* promastigotes were stained separately with PKH26 and PKH67 dyes, respectively. Dual staining enabled detection of each cell type, where non-infected macrophages were also recorded for the quantification. Dual-PKH staining achieved high success in selective staining of promastigotes (99.71%) and macrophages (99.57%). The percentages of parasite-infected macrophages were determined for initial 1:2.5 and 1:10 infection ratios as 15.68 and 61.70%, respectively; indicating significant increase in infection rate parallel to the initial treatment ratio. These results demonstrated that the introduced dual-fluorescence flow cytometric approach can be successfully used as an accurate and rapid quantification method for *L. infantum*-infected macrophages and strengthens the hypothesis that flow cytometric approaches could replace conventional microscopic methodologies.

## Graphical abstract

**Figure:**
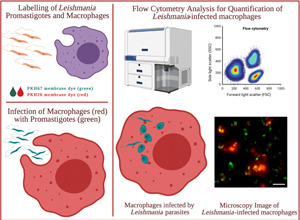

## Introduction

Leishmaniasis is a neglected tropical disease caused by the vector-borne parasites of the genus *Leishmania*, transmitted to numerous species including humans through the bite of a sand fly, mainly Phlebotomus and Lutzomyia. There are over 20 species of *Leishmania* that cause life-threatening disorders widely distributed in 98 tropical and subtropical regions including Asia, South America, Northern Africa, Southern Europe, the Middle East and Turkey. According to the recent WHO report, more than 350 000 people estimated at risk and 1.3 million new cases of leishmaniasis occur every year (Gradoni *et al*., 2017). *Leishmania* spp. are digenetic organisms which survive and replicate either as promastigotes in an insect vector, or as amastigote forms within phagolysosome-like vacuoles inside the macrophages (Sereno *et al*., 2007; Arenas *et al*., 2017). Clinical manifestations of leishmaniasis are divided into three clinical forms: cutaneous, mucocutaneous or visceral leishmaniasis known as ‘Kala Azar’, depending on which species are involved in the infection within mammalian tissues.

The molecular mechanisms that provide the control of the infection and the development of new treatment strategies, are critical for understanding of the invasion and manipulation of *Leishmania* species to host cells (Haridas *et al*., 2017). Cellular factors that identify the intracellular interaction of parasites with the macrophages are traditionally determined by the quantification of cellular population (Gradoni *et al*., 2017; Haridas *et al*., 2017). The available experimental techniques include conventional microscopic analysis and alternative flow cytometry approach for the determination of the infection rates and the antileishmanial activities for the tested drugs, based on the quantification of intracellular parasite population in each host cell (Ogunkolade *et al*., 1990; Bertho *et al*., 1992; Di Giorgio *et al*., 2000; Guinet *et al*., 2000; Sereno *et al*., 2007; Haridas *et al*., 2017). As the most common approach, microscopy analysis is widely used for the quantification of adherent infected macrophages (Berman *et al*., 1979; Di Giorgio *et al*., 2000; Guinet *et al*., 2000; van Zandbergen *et al*., 2004; Sereno *et al*., 2007; Bettencourt *et al*., 2010; Haridas *et al*., 2017; Zheng *et al*., 2020), where microscopic images were investigated for the presence of parasites inside the host cell. However, as the intracellular parasites replicate heterogeneously within each host cell, the number of parasites in each macrophage varies for each cell. In addition, a complete analyses of the infection necessitates quantification of parasites from thousands of cellular images in a short period of time (Muskavitch *et al*., 2008; Walker *et al*., 2013; Haridas *et al*., 2017), where the microscopy technique becomes insufficient with regard to time and effort. Alternatively, fluorescence-based assays have been developed for the investigation of interactions between parasite and host systems (Bertho *et al*., 1992; Azas *et al*., 1997; Guinet *et al*., 2000; Sereno *et al*., 2007; Millington *et al*., 2010; Haridas *et al*., 2017). As previously suggested, flow cytometry technique provides the accurate and reliable semi-quantitative analysis of thousands of cells in a short period of time under automation system, where based on the different morphological features of the cell populations direct visualization of fluorescently labelled macrophages and parasites were enabled as events on dot plots, thereby allowing reliable quantification of infected macrophages as well as number of intracellular parasites per host cell indicating the severity of the infection (Ogunkolade *et al*., 1990; Bertho *et al*., 1992; Azas *et al*., 1997; Di Giorgio *et al*., 2000; Guinet *et al*., 2000; Sereno *et al*., 2007; Haridas *et al*., 2017).

As cell detection markers, fluorescent lipophilic PKH dyes such as PKH26 (red-fluorescence dye) and PKH67 (green-fluorescence dye) are frequently used to label cell membranes due to their high selectivity and sensitivity (Poon *et al*., 2000; Fischer and Mackensen, 2003; Tario *et al*., 2012). Through strong noncovalent interactions, the aliphatic tail of these dyes rapidly intercalates into the lipid bilayer, promoting long-term dye retention and stable fluorescence intensity (Pužar Dominkuš *et al*., 2018). On the other hand, PKH dyes display stable, relatively nontoxic properties and do not cause any alteration in the cell functions, as their partitioning occurs inside the lipid region of cell membranes rather than the surface proteins (Horan and Slezak, 1989; Horan *et al*., 1990; Wallace *et al*., 1993; Raybourne and Bunning, 1994; Taguchi *et al*., 1995; Hara-Kaonga and Pistole, 2007). In addition, PKH dyes offer a rapid and efficient staining procedure for monitoring and detection for the cell uptake of microorganisms (Raybourne and Bunning, 1994; Abdullah *et al*., 1999; Hara-Kaonga and Pistole, 2007), exosomes (Pužar Dominkuš *et al*., 2018; Shimomura *et al*., 2020) and various nanoparticles by flow cytometry and fluorescent microscopy both *in vitro* and *in vivo* (Parish, 1999; Bapat *et al*., 2010; Schubert *et al*., 2011; Pužar Dominkuš *et al*., 2018). For instance, PKH lipid membrane dyes have been previously applied to studies in the quantification of internalized microorganism such as bacteria and *Leishmania* species within the mammalian host cells (Abdullah *et al*., 1999; Hara-Kaonga and Pistole, 2007). Abdullah *et al*. (1999) suggested prior staining of *Leishmania infantum* promastigotes with PKH fluorescent dye before the macrophage infection, which further allowed accurate detection of cell infection (Abdullah *et al*., 1999). Improving this approach, we applied the staining on both macrophages and promastigotes simultaneously using PKH26 and PKH67 membrane dyes, respectively. The independent emission spectra of the dyes were expected to enable real-time detection of both PKH26-labelled macrophages and PKH67-labelled *L. infantum* parasites without overlapping and establishes the basis of the research.

Hence, this paper describes in detail the applied procedure for the determination of infection rates of macrophages infected with *L. infantum* using flow cytometry. Prior the infection, the macrophages and the parasites were stained separately by PKH26 and PKH67 dyes, respectively, which enabled detection of each cell type by flow cytometry. The success of the selectivity in staining the cells was evaluated and verified through co-culture inspections under fluorescence microscopy. The procedure introduced in this paper yields quantification of *Leishmania*-infected macrophages as percentage of infection (%), where the non-infected macrophages were also recorded for the quantification.

## Materials and methods

### Materials

*Leishmania infantum* promastigotes (MHOM/MA/67/ITMAP-263) were kindly provided by Dr Ana Tomás [Institute for Molecular and Cell Biology (IBMC), University of Porto (Portugal)]. The murine macrophage cell line J774A.1 (TIB-67) was obtained from American Type Culture Collection (ATCC) (Rockville, MD). PKH67 Green Fluorescent Cell Linker Kits and PKH26 Red Fluorescent Cell Linker Kits were purchased from Sigma–Aldrich, Germany.

### Parasite and macrophage cell culture

#### Parasite culture

*Leishmania infantum* promastigotes were grown at 25°C in RPMI 1640 Glutamax (Gibco) supplemented with 10% (v/v) inactivated foetal bovine serum (iFBS), 50 U mL^−1^ penicillin, 50 *μ*g mL^−1^ streptomycin, and 20 mm HEPES sodium salt pH 7.4 (Sigma Aldrich, Germany). To maintain infectivity of parasites in culture, parasites were kept for no more than seven passages in culture flask. Infection experiments were performed with recently thawed aliquots of virulent parasites. Infective metacyclic promastigotes were obtained from stationary phase cultures of *L. infantum*. After centrifugation at 3000 × ***g*** for 10 min, promastigotes were collected from the pellet (Gomes-Alves *et al*., 2018).

### Macrophage cell culture

J774A.1 (TIB-67) macrophages were cultured in Dulbecco' modified Eagle' medium (DMEM) supplemented with 10% inactivated foetal bovine serum (FBS) (Invitrogen), 2 mm L-glutamine, 100 units mL^−1^ of penicillin and 100 *μ*g mL^−1^ of streptomycin (Biological Industries, Beit Haemek, Israel) in plastic flasks. Cells were maintained in culture by sub-passaging every 3 days.

### Staining of macrophages and parasites with fluorescent dyes

J774A.1 macrophages and *L. infantum* promastigotes were stained with PKH26 dye (Red Fluorescent Cell Linker Kit, Sigma-Aldrich) and PKH67 dye (Green Fluorescent Cell Linker Kit, Sigma-Aldrich), respectively. The manufacturer' protocols were followed for staining. Briefly, a total of 10^7^ cells mL^−1^ were centrifuged (400 × ***g***) for 5 min into a loose pellet. Pellet was collected and resuspended with 1 mL of diluent to prepare a 2× cell suspension. Prior the staining, 2× dye solution (4 × 10^−6^ M) in Diluent C by adding 4 *μ*L of the PKH ethanolic dye solution to 1 mL of Diluent C were prepared in a falcon tube. Afterwards, 1 mL of 2× dye solution were mixed with 1 mL of 2× cell suspension to obtain 1 × 10^7^ cells mL^−1^ and 2 × 10^−6^ M PKH26 dye. After incubation of the cell/dye suspension for 1–5 min, the staining was stopped by adding an equal volume (2 mL) of serum and incubated for 1 min to allow binding of excess dye. Cells were washed following two more centrifugation steps (400 × ***g*** for 10 min) to ensure removal of unbound dye and then resuspended in a complete medium. Cells were fixed in 2% paraformaldehyde by treatment for 15 min. Prior the infection, the promastigotes and macrophages were examined by flow cytometry (CytoFLEX S, Beckman Coulter, USA) with a total of 100 000 events to ensure that all cells are successfully stained with PKH dyes and can be detected.

### Flow cytometry for cell viability analysis of macrophages infected with *Leishmania infantum* promastigotes

J774A.1 macrophages stained by PKH26 were seeded on 12-well plates with a density of 180 000 cells per well and incubated at 37°C. After 24 h, J774A.1 cells were infected with stationary-phase PKH67-labelled promastigotes (cell:promastigote ratio, 1:2.5 and 1:10). Following 3-h of co-incubation, non-internalized parasites were removed by successive washing with PBS. After 24 h, cells were fixed with 2% paraformaldehyde, and then washed three times with PBS. Infection rates (%) of macrophages were determined according to their relative fluorescence intensities detected by flow cytometry (CytoFLEX S, Beckman Coulter, USA).

For the detection of PKH-labelled parasites/cells, FL1 (green fluorescence) and FL2 (red fluorescence) filters were used to distinguish the parasites/cells with regard to the excitation (for PKH67 at 490 nm; PKH26 at 551 nm) and emission (for PKH67 at 502 nm; PKH26 at 567 nm) of the dyes. Reliability of flow cytometry dot-plot data were verified by performing six independent experiment sets. Gates were set for each independent experiment to optimize the separation of infected cells. At least 100 000 cells were collected and read on flow cytometry to extend the reliability of the statistical data. In parallel, fluorescence microscopy images were taken to visualize the parasite infection by the use of different fluorescent channels to distinguish PKH67-labelled parasites (green), PKH26-labelled macrophages (red) and infected macrophages with a merged yellow colour.

### Statistical analysis

Statistical analysis was performed on GraphPad Prism Software (version 6.01). The data sets were compared using an unpaired *t*-test (also known as an independent *t*-test). Differences were considered statistically significant at (*) *P* ⩽ 0.05, (**) *P* ⩽ 0.01, (***) *P* ⩽ 0.001, (****) *P* ⩽ 0.0001.

## Results

### Cell culture studies

#### Staining of the parasites and macrophages with PKH membrane dyes

**Fig. 1.**
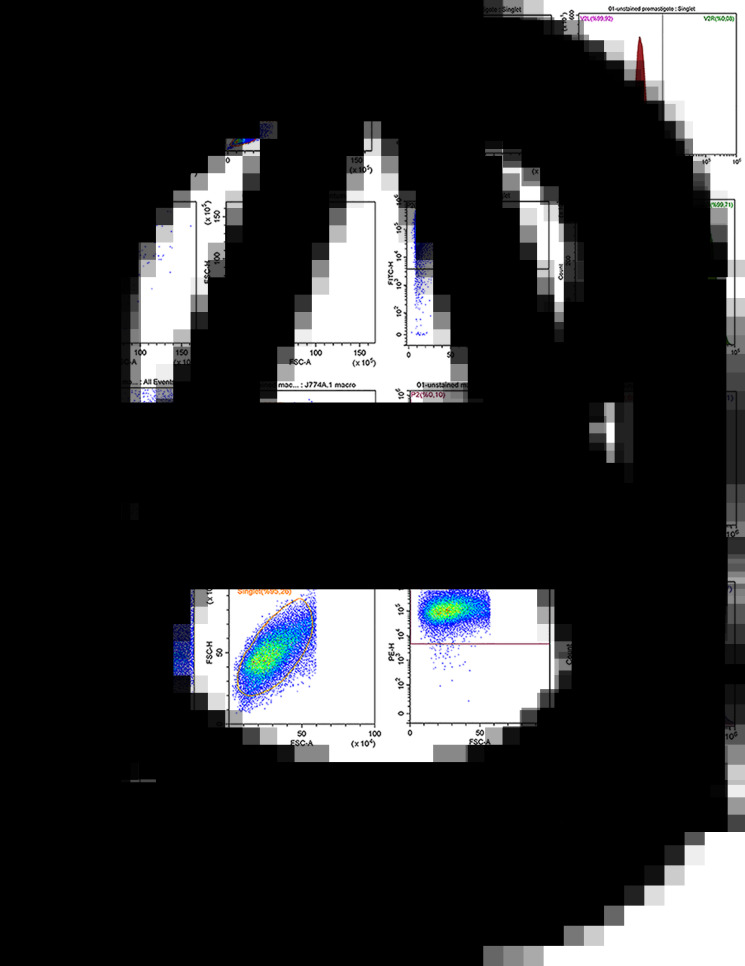
Flow cytometry analysis of PKH67-stained and unstained *Leishmania infantum* promastigotes. Flow cytometric (pseudo-colour/smooth) plots for (A) unstained and (B) PKH67-stained *L. infantum* promastigotes; (C) unstained and (D) PKH26-stained J774 A.1 macrophages.

J774A.1 macrophages and *L. infantum* parasites were stained with PKH26 (red) and PKH67 (green) dyes, respectively. Tracking the green FITC signal (PKH67) and the red PE signal (PKH26) in flow cytometry, it was possible to determine separately the rates of PKH67-staining on *L. infantum* promastigotes and PKH26-staining on J774 A.1 macrophages. Accordingly, flow cytometric (pseudo-colour/smooth) plots indicated the gating strategy based on the size and granularity of *L. infantum* promastigotes and J774 A.1 macrophages using a minimum/maximum SSC-A/FSC-A filter level to exclude debris and cluster of the cells (Fig. 1). Cells were gated to identify the distinct area for the cell population. Within the group of gated cells, single cells (singlets) were sub-gated by considering their FSC-H/FSC-A features. As illustrated in Fig. 1A, when unstained parasites were analysed in flow cytometry with this procedure, 99.92% of parasites were determined as unstained, where 0.08% of the parasites were left out beyond the constraints owing a false FITC signal. When stained parasites were analysed in flow cytometry with the same procedure, single cells or so-named singlets corresponding to 99.32% of the population were plotted with the PKH67 green fluorescence dye, of which 99.71% of promastigote population was found to be labelled successfully (Fig. 1B). The unstained macrophage population was detected as 99.99% by flow cytometry, while singlets of PKH26-stained macrophages corresponded to 95.26% of the population and 99.57% of this singlet population was found to be labelled successfully (Fig. 1C, D). These findings indicate that a negligible number of promastigotes remain unstained subsequent to the applied staining protocol.

### Quantification of the infection rate (%) of Leishmania-infected macrophages

**Fig. 2.**
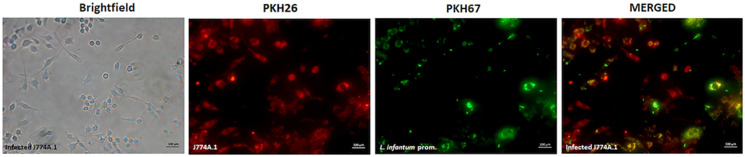
Fluorescence microscopy images of macrophages infected with *Leishmania infantum* promastigotes (infection ratio, 1:10). From left to right: brightfield image, fluorescence image of PKH26-stained macrophages (red), PKH67-stained *L. infantum* promastigotes (green), and *Leishmania*-infected macrophages (merged – yellow) (magnification: 20×). Scale bars correspond to 100 *μ*m.

In order to investigate the infection rates of macrophages, staining was applied this time on both *L. infantum* promastigotes and J774A.1 macrophages with PKH67 (green) and PKH26 (red) dyes, respectively. While the tracking of the fluorescent signals in fluorescence microscopy enabled the selective detection of promastigotes, non-infected macrophages (the macrophages without parasite inside) and infected macrophages qualitatively, the flow cytometry allowed quantitative analysis of the cells and infection ratio of the macrophages. Accordingly, the fluorescence microscopy images displayed promastigotes in green, non-infected macrophages in red, and the macrophages infected by the parasites as the merged yellow colour (Fig. 2). The bright-field image confirmed that all macrophages and promastigotes have been labelled and detected by fluorescence microscopy imaging. In addition to the determination of the infection, microscopy analysis also verified that all macrophages could be successfully stained by the applied protocol, as during multi-channel reconstruction of the bright-field and fluorescent channel images no macrophage was observed lacking the red fluorescence signal.

**Fig. 3.**
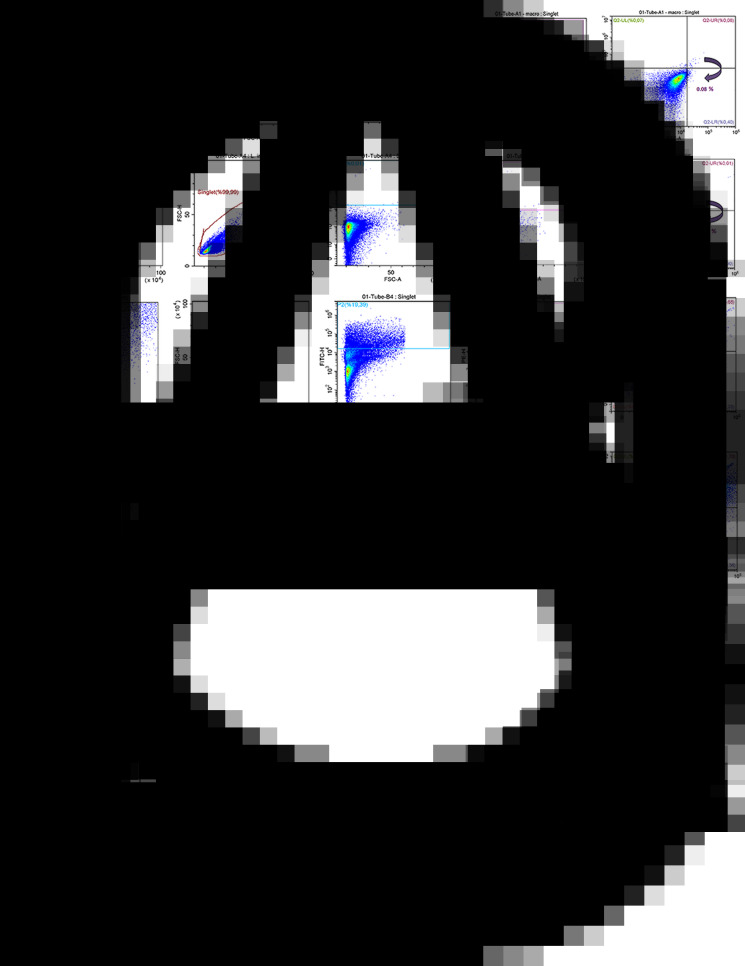
The flow cytometric (pseudo-colour/smooth) plots of (A) unstained non-infected macrophages, (B) unstained promastigotes and (C and D) *Leishmania*-infected macrophages at 1:2.5 and 1:10 rates of infection, respectively.

Flow cytometric (pseudo-colour/smooth) analysis enabled the quantitative determination of the percentage of macrophages infected with *L. infantum* promastigotes. For this analysis, J774A.1 macrophage culture was infected for 3 h with *L. infantum* promastigotes by two different macrophage:parasite ratios, i.e. 1:2.5 and 1:10. The flow cytometry analysis started first with unstained promastigote and macrophage (not infected) samples. The obtained FSC-H/FSC-A cytograms indicated a homogenous population distribution for both unstained macrophages (Fig. 3A) and unstained promastigotes (Fig. 3B). When the means of fluorescence intensities of a FL-1 green (FITC) and a FL-2 (PE) signals were investigated, as expected, no fluorescence signal was detected for unstained cells. On the other hand, *Leishmania*-infected macrophages were gated based on the information on cytogram for non-infected macrophage populations (Fig. 3C, D) and could be compensated using PKH26-stained macrophages and PKH67-stained promastigotes (Fig. 1). From the gated population of infected macrophages, singlets were sub-gated considering their FSC-H/FSC-A cytograph. This way, fluorescent signal of PKH26-stained macrophage population was successfully separated from background fluorescence of the macrophages (Fig. 3). Singlets were plotted according to the PKH67 green and PKH26 red fluorescence signals using quadrant analysis, where infected macrophages were identified by their double positive signal in the upper-right quadrant using FITC and PE filter (Fig. 3C, D). Further quadrants represent non-infected macrophages and non-internalized promastigotes. In detail, PKH26-stained non-infected macrophages (with high PE signal but low FITC signal) are plotted in upper-left, whereas PKH67-stained non-internalized promastigotes (with low PE signal but high FITC signal) are plotted in lower-right on the cytograms. When the populations in the lower-left and upper-right quadrants in Fig. 3C and D were analysed together, a significant increase (*P* ⩽ 0.0001) in infection rate of macrophages could be observed in parallel to the increase in the initial treatment ratios. Accordingly, the macrophage populations became 15.68 and 61.70% infected by *L. infantum* parasites, when incubated with 1:2.5 and 1:10 macrophage:promastigote ratios, respectively.

Flow cytometry data also suggested a higher degree granularity at infected macrophages parallel to the initial parasitic load (Fig. 3C, D). Moreover, when the macrophages are infected with higher initial parasite load, an increase is observed in SSC-signal intensity **(**Fig. 3D; SSC-FSC cytogram), which is attributed to the increase in parasite population inside the macrophages.

## Discussion

Flow cytometry analysis emerges as an alternative methodology to determine the rates of *Leishmania*-infected macrophages rapidly and accurately. Prior any investigation, the proposed procedure complies fluorescent staining of macrophages and *L. infantum* parasites separately by PKH26 and PKH67 lipophilic dyes, respectively. (Fluorescence microscopy analysis is used to verify the staining of each parasite and macrophage). Distinctive fluorescence signals emitted by the macrophage and the parasite enables the detection of uninfected and infected macrophages, where the fluorescent signal only from PKH26 indicates no presence of parasites inside the macrophage, while the merged fluorescent signal of PKH26 and PKH67 implies the occurrence of infection. The analysis allows the determination of infection of macrophages as percentage in total macrophage population. The procedure is fast and straightforward, once the configurations of laser and the borders of the gating are correctly set for the fluorescent-labelled macrophages and parasites. The sensitivity of the applied methodology is high to determine the presence of parasite inside the macrophages, yet the technique does not allow to estimate the number of parasites within each macrophage, which might be important to further assess the severity of the infection together with the percentage of macrophage infection. Thus, the introduced fluorescence-based methodology provides rapid analysis of higher numbers of cells than the alternative microscopic approaches can provide, and could be suggested as a more suitable approach for the evaluation of the potency of antileishmanial drug candidates (Ogunkolade *et al*., 1990; Bertho *et al*., 1992; Azas *et al*., 1997; Di Giorgio *et al*., 2000; Guinet *et al*., 2000; Sereno *et al*., 2007; Haridas *et al*., 2017). Indeed, flow cytometry was already reported as a tool to investigate macrophage infection previously by multiple studies, where the aims of the researchers were evaluation of either antibacterial or antiparasitic activity of the tested drug molecules (Ogunkolade *et al*., 1990; Bertho *et al*., 1992; Azas *et al*., 1997; Di Giorgio *et al*., 2000; Guinet *et al*., 2000; Sereno *et al*., 2007; Haridas *et al*., 2017). Among them, Abdullah *et al*. (1999) suggested a procedure, wherein *L. infantum* promastigotes were stained with PKH2-GL fluorescent dye before macrophage infection, which allowed accurate detection of cell infection (Abdullah *et al*., 1999); however, could not detect intracellular amastigotes after prolonged incubation periods. Likewise, Di Giorgio *et al*. (2000) suggested labelling of intracellular amastigotes with *Leishmania* lipophosphoglycan-specific monoclonal antibody after macrophage infection (Di Giorgio *et al*., 2000). That technique allowed accurate quantification of infection, but failed at detection of non-infected macrophages specifically. Therefore, the novel strategy including the labelling of both macrophages and promastigotes by two distinctive fluorescent dyes (PKH26 red and PKH67 green, respectively) improved the detection capacity of the flow cytometry approach and distinguished the current study from the previous approaches.

Since the approach involves the simultaneous use of PKH26 and PKH67, certain modifications were also necessary in the applied flow cytometry procedure. An analysis gate on an FSC *vs* SSC scatter plot was defined prior the flow cytometry analysis, where this graphical boundary defined the characteristics of cells to include for the analysis according to the size and internal complexity. Next, dual labelling necessitated FL1/FL2 dual-colour fluorescence gating, which allowed distinction of intracellular parasites from free parasites where the two-colour gating region identifies infected macrophages. Proportion of the infected macrophages in the total cell population can be precisely determined by the use of gates or quadrants. Thereby providing understanding on the level of infection and the success rate of the antileishmanial therapies tested.

Dual-PKH staining achieved high success rate in selective staining of the promastigotes (99.71%) and macrophages (99.57%) as verified by both fluorescence microscopy (Fig. 2) and flow cytometry analysis (Fig. 1). A negligible number of promastigotes were observed to remain unstained (Fig. 1). In the flow cytometric (pseudo-colour/smooth) plots, events were gated according to the total parasite population (Fig. 1). The singlet promastigote population corresponded to 99.32% of the total parasite population, and 99.71% of the singlet population was found to be labelled with PKH67 green fluorescence dye as indicated by plot of FITC fluorescent channel (Fig. 1B). The unstained macrophage population was detected as 99.99% by flow cytometry, while singlets of PKH26-stained macrophages corresponded to 95.26% of the population and 99.57% of this singlet population was found to be labelled successfully (Fig. 1C, D).

As illustrated in Fig. 3, *Leishmania*-infected and non-infected cells could be separated into two well-isolated populations using the flow cytometric quadrant analysis, which not only allowed quantitative estimation of infection rates (%) of macrophages, but also enhanced a discrimination of noninfected macrophages. The percentage of parasite-infected macrophages with infection ratio of 1:2.5 and 1:10 was determined as 15.68 and 61.70%, respectively (Fig. 3C, D). Infection rates of the macrophages were found to increase in parallel to the initial treatment ratios as revealed by mean fluorescence intensity of double-positive macrophages, and the difference between the infection rates were found to be statistically significant (*P* ⩽ 0.0001).

The quantitative results of flow cytometry analysis were supported by fluorescence microscopy analysis (Fig. 2). Providing qualitative information on the infection rates, fluorescence microscopy displayed the macrophages (red) infected by *L. infantum* parasites (green) with the merged yellow colour. Fluorescence microscopy analysis also showed that PKH staining did not cause any dye-induced cell death on both macrophages and *L. infantum* promastigotes as supported by the literature (Li *et al*., 2013). These findings suggest that PKH26 and PKH67 are safe to be used for macrophages and *L. infantum* parasites, while some toxicity problems have been reported for PKH2-GL staining on *L. donovani*, *L. major* and *L. infantum* parasites and macrophages infected therewith (Abdullah *et al*., 1999). The lower infection rates in *L. infantum*-infected macrophages was explained in linked with the toxicity effect of PKH2-GL, which can be seen as reduced forward scatter in the dot plot (Abdullah *et al*., 1999). Contrary, in our study the high infection rates up to 61.70% indicated that the PKH26 and PKH67 dyes can remain intact within the cell membrane and are safe for use at least for 24-h incubation periods.

Overall, the findings of this study introduce the applied flow cytometry procedure as a reliable approach to quantify *Leishmania* infection in J774 A.1 macrophages infected with *L. infantum* parasites with high specificity. Dual-coloured fluorescence gating for PKH26-stained macrophages and PKH67-stained *L. infantum* promastigotes in flow cytometric approach emerges as a better alternative to the conventional microscopic approach, in particular for the analysis of large macrophage populations with *L. infantum* infections in a shorter period of time.

## Conclusion

On the basis of these results, the proposed flow cytometric approach illustrates an accurate and relatively rapid quantification for the determination of the infection rates (%) of macrophages with *L. infantum* parasites. The use of PKH26 and PKH67 fluorescent membrane dyes allows selective labelling of macrophages and parasites, respectively. High rate of success in distinctive dual-labelling and the high sensitivity in detection of infection by flow cytometry strengthens the hypothesis that the flow cytometric approach could replace the conventional microscopic methodologies that are stuck for speed and accuracy in detection of infected macrophages. The ease of application and the reproducibility of the collected data constitute the other advantages of the technique.

## Data

The raw data supporting the conclusions of this article will be made available by the authors, without undue reservation, to any qualified researcher.
